# Umbilical cord blood therapy modulates neonatal hypoxic ischemic brain injury in both females and males

**DOI:** 10.1038/s41598-021-95035-1

**Published:** 2021-08-04

**Authors:** Tayla R. Penny, Yen Pham, Amy E. Sutherland, Joohyung Lee, Graham Jenkin, Michael C. Fahey, Suzanne L. Miller, Courtney A. McDonald

**Affiliations:** 1 The Ritchie Centre, Hudson Institute of Medical Research, 27-31 Wright St, Clayton, VIC 3168 Australia; 2 Department of Obstetrics and Gynaecology, Monash University, Clayton, VIC Australia; 3 Centre for Endocrinology and Metabolism, Hudson Institute of Medical Research, Clayton, Australia; 4 Department of Paediatrics, Monash University, Clayton, VIC Australia

**Keywords:** Hypoxic-ischaemic encephalopathy, Preclinical research, Stem-cell research

## Abstract

Preclinical and clinical studies have shown that sex is a significant risk factor for perinatal morbidity and mortality, with males being more susceptible to neonatal hypoxic ischemic (HI) brain injury. No study has investigated sexual dimorphism in the efficacy of umbilical cord blood (UCB) cell therapy. HI injury was induced in postnatal day 10 (PND10) rat pups using the Rice-Vannucci method of carotid artery ligation. Pups received 3 doses of UCB cells (PND11, 13, 20) and underwent behavioural testing. On PND50, brains were collected for immunohistochemical analysis. Behavioural and neuropathological outcomes were assessed for sex differences. HI brain injury resulted in a significant decrease in brain weight and increase in tissue loss in females and males. Females and males also exhibited significant cell death, region-specific neuron loss and long-term behavioural deficits. Females had significantly smaller brains overall compared to males and males had significantly reduced neuron numbers in the cortex compared to females. UCB administration improved multiple aspects of neuropathology and functional outcomes in males and females. Females and males both exhibited injury following HI. This is the first preclinical evidence that UCB is an appropriate treatment for neonatal brain injury in both female and male neonates.

## Introduction

Neonatal hypoxic ischemic encephalopathy (HIE) is a condition whereby a lack of blood and oxygen during gestation or at birth causes injury to the brain^1^. HIE is a major cause of lifelong motor and cognitive impairments, affecting approximately 2 in 1000 term births in developed countries^2^. Of these, approximately 60% of untreated neonates will die or suffer from subsequent long-term neurodevelopmental conditions such as cerebral palsy (CP), epilepsy or learning disabilities^1,3^. HIE and CP associated with HIE show a gender bias where males account for a higher percentage of cases than females^4^. This phenomenon is referred to as ‘the male disadvantage’^5–7^. In a clinical study assessing different risk factors associated with HIE, males had a 1.27-fold higher incidence of HIE compared to females^8^. In addition, males account for 56–58% of CP cases in Australia^6^ and the incidence of CP in Europe is 30% higher in males^9^.

Preclinical studies have previously investigated the sexual dimorphism observed in HIE and CP using animal models; however, the preclinical findings do not always reflect the level of sexual dimorphism in the clinical condition. Studies that have utilised the term-equivalent Rice-Vannucci rodent model of neonatal hypoxic ischemic (HI) brain injury^10^ have shown that males and females both exhibit adverse neuropathologies and functional deficits in response to HI, but the mechanisms driving the progression of injury may differ and be sex specific^11^. In terms of behavioural outcomes, there is no consensus about whether males or females show a greater degree of disadvantage, although some preclinical studies have demonstrated that, overall, neurodevelopmental outcomes, motor function and sensory function, do not exhibit sex differences^11^. However, it has been shown, that male rodents exhibit worse outcomes in early motor function tests such as the negative geotaxis and wire suspension tests^12^. Further, preclinical studies have shown that learning and memory deficits may also be sex biased. Female offspring exhibit a greater deficit in tasks that assess spatial memory such as the water maze^13–17^. In contrast, males showed a significant deficit in short-term memory recall in novel object recognition tests^12,18^.

In response to HI there are also disparate neuropathology outcomes in males and females. Studies have shown that males are more susceptible to neurodegeneration in specific structures such as the cortex^19^ or the hippocampus in preterm^20^ and term^21^ neonates, whereas females are more vulnerable to hemispheric tissue loss^15^. However, this is not a consistent observation, as some studies have shown no difference in tissue loss between sexes^16,20,22–24^. Males and females are both known to exhibit significant neuroinflammation following a HI insult, with possible sex differences in local and systemic inflammation^25,26^. Males appear to be more susceptible to oxidative stress than females, leading to the production of reactive oxygen species (ROS), which are upstream regulators of the caspase-independent cell death pathway, the most common pathway of neuronal cell death in males^27,28^. Conversely, it has been shown that the caspase-dependent pathway is the dominant mechanism of neuronal cell death in females following a HI injury^27,28^. Investigating potential therapies which target all mechanisms known to be upregulated following a HI insult in both males and females is essential.

Therapeutic hypothermia is currently the only standard neuroprotective treatment for term infants diagnosed with HIE. Whether hypothermia is equally neuroprotective in males and females remains unknown^29^, however preclinical rodent studies have suggested it is more protective in females^29–31^. Other neuroprotective agents have also shown sex-specific effects, for example the antioxidant N-Acetylcysteine improved short-term structural outcomes in females only, but improved long-term functional outcomes in both sexes^32^. Erythropoietin (EPO) is a neuroprotective therapy for neonates at clinical trial stage, and is more effective in reducing brain infarcts in females than males, and improving long-term sensorimotor function in a preclinical rodent model of stroke^33^. Given the potential for sex-specific effects, it is important to consider whether potential neuroprotective agents are effective in both males and females, to ensure effective clinical translation.

Umbilical cord blood (UCB) improves neuropathology and behavioural outcomes in both preclinical studies and human clinical trials of HIE and CP^34^. Specifically, preclinical studies in rodents have demonstrated that UCB reduced caspase mediated apoptosis^35–39^, neuroinflammation^35–38,40^ and tissue loss^35,41^ following neonatal HI insult. UCB administration also improves short and long-term behavioural outcomes^35,37,39,40,42–49^. In this study, we have investigated both the behavioural and neuropathological efficacy of UCB cells in males and females as a potential therapy for HIE. We hypothesised that UCB would modulate brain injury in both males and females, thus showing no sexual dimorphism in therapeutic efficacy.

## Results

A total of 91 rat pups (from 13 dams) were used in this study. 66 animals were exposed to HI (HI, n = 15M, 19F; UCB, n = 16M, 16F) and 25 animals received sham surgery with no HI (Sham; n = 10M, 15F). The animals used in this study were part of a larger cohort which had an overall mortality rate during surgery/hypoxia of 3.9% (5/129; 2 males, 3 females). Following allocation to treatment groups, one pup died from the UCB group (1 female), and no pups died from the HI and Sham groups.

### Behavioural outcomes

#### Negative geotaxis

**Figure 1:**
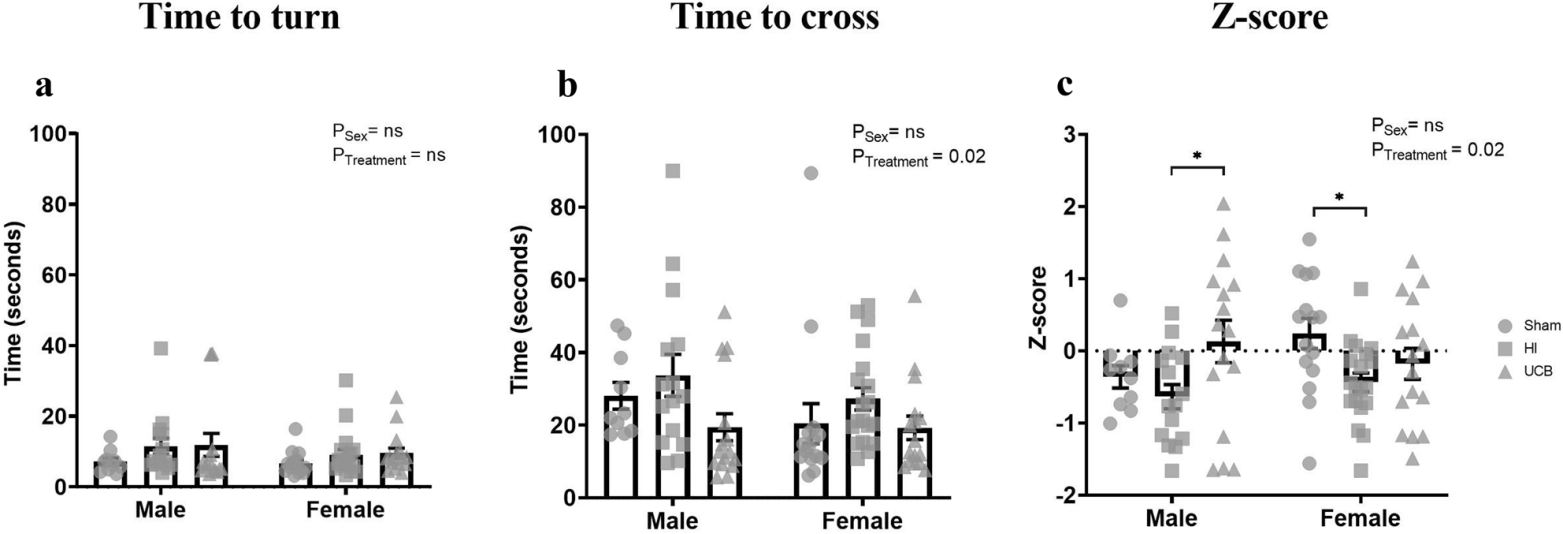
Raw and transformed scores for negative geotaxis analysis. The time taken to turn 180° on the angled board (**a**) and walk up the angled board (**b**) on PND14. Raw scores were transformed into a negative geotaxis Z-score (**c**). A positive Z-score reflects an improvement in behaviour, negative Z-score reflects deficit in behaviour. (In these graphs, the sham group is represented by a circle, the HI group is represented by a square and the UCB groups is represented by a triangle. Data expressed as mean ± SEM, n = 10–19 pups per group, *P < 0.05).

The negative geotaxis test evaluates strength and coordination, as well as the innate vestibular reflex. Overall, there was no effect of sex seen in the time to turn or time to cross the line (Fig. 1a,b), and an overall treatment effect was seen in the time to cross the line (Fig. 1b; P = 0.02). Multiple comparisons show that males and females showed no difference between groups for both time to turn and time to walk up the angled board (Fig. 1a,b). These scores were also transformed into z-scores for standardisation. A positive Z-score is indicative of an improved behavioural outcome, while a negative score is indicative of a worsened behavioural outcome. Overall, there was no effect of sex seen in the Z-score, however there was a significant treatment effect (Fig. 1c; P = 0.02). For the negative geotaxis test Z-score, there was no difference in males in the sham and HI groups, however the UCB treated group exhibited a significant improvement in behaviour compared to the HI group (Fig. 1c; P = 0.02). HI Females demonstrated a significant behavioural deficit compared to sham (Fig. 1c; P = 0.044), however UCB did not significantly improve this behavioural deficit.

#### Novel object recognition

**Figure 2:**
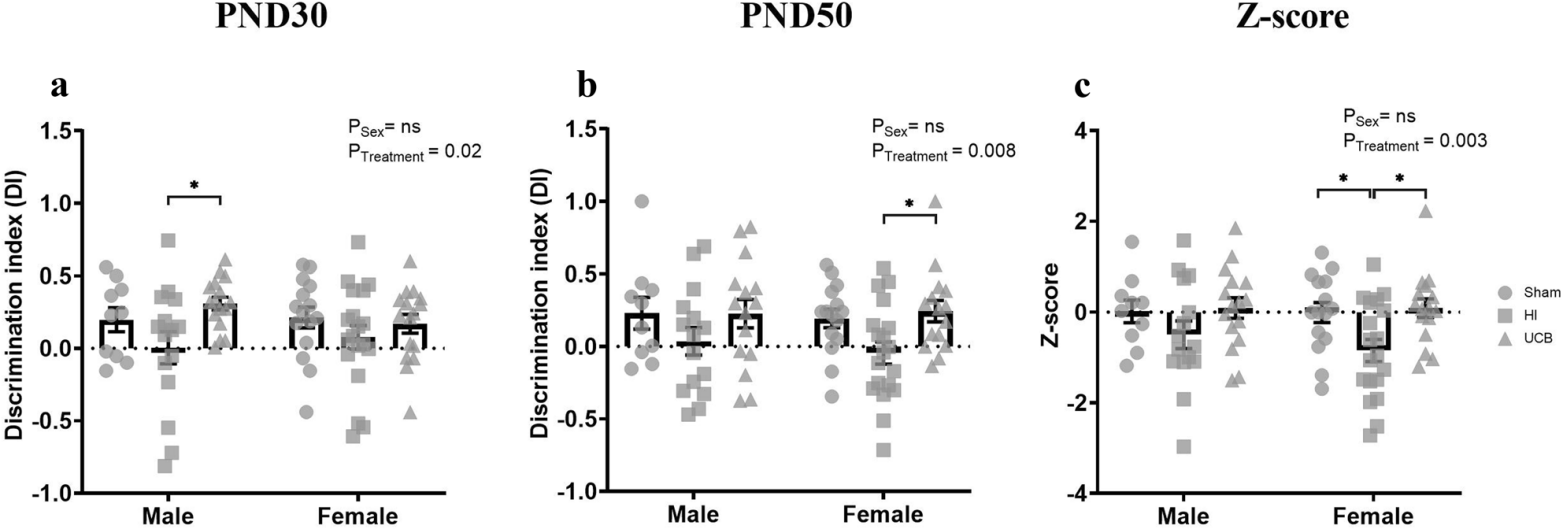
Raw and transformed scores for the novel object recognition test. Discrimination index (DI) was calculated for PND30 (**a**) and PND50 (**b**) where a positive DI shows preference toward the novel object and a negative score shows preference toward the familiar object. Raw scores were transformed into a novel object recognition Z-score (**c**). A positive Z-score reflects an improvement in behaviour, negative Z-score reflects deficit in behaviour. (In these graphs, the sham group is represented by a circle, the HI group is represented by a square and the UCB groups is represented by a triangle. Data expressed as mean ± SEM, n = 10–19 pups per group, *P < 0.05).

The novel object recognition test was used to assess short-term memory and exploratory behaviours. The data from this test was presented as a discrimination index (DI) where a positive DI shows preference toward the novel object and a negative DI shows preference toward the familiar object. On Postnatal day (PND30), there was an overall effect of treatment (Fig. 2a; P = 0.02), however there was not an overall effect of sex (Fig. 2a). In addition, there was no significant difference between sham and HI in males, however the sham group showed a preference toward the novel object and the HI group did not show a preference to either object (Fig. 2a). The UCB group exhibited a significant positive preference compared to the HI group (Fig. 2a; P = 0.019). On PND30, there were no differences between the groups in females (Fig. 2a).

On PND50, there was an overall treatment effect (Fig. 2b; P = 0.008), however there was no effect of sex overall (Fig. 2b). Males did not demonstrate a significant difference between groups in the novel object recognition test, however they did show a trend towards a negative preference in the HI group compared to sham, and a trend towards a positive preference in the UCB group compared to HI (Fig. 2b). Females in the HI group exhibited a trend towards a negative preference compared to the sham group, and the UCB group showed a significant positive preference (toward the novel object), compared to the HI group (Fig. 2b; P = 0.033).

DI from PND30 and 50 was transformed into Z-scores and combined to demonstrate short-term memory deficits throughout the experiment. When transformed into Z-scores, sex did not show an overall effect, however the treatment effect was significant (Fig. 2c; P = 0.003). Males showed no significant difference between groups; however, the HI group was the only group to have a negative Z-score which is indicative of worse outcomes (Fig. 2c). The females, had a significantly decreased Z-score in the HI group compared to sham (Fig. 2c; P = 0.034) and the Z-score in the UCB group was significantly increased compared to HI in females (Fig. 2c; P = 0.014).

#### Cylinder test

**Figure 3:**
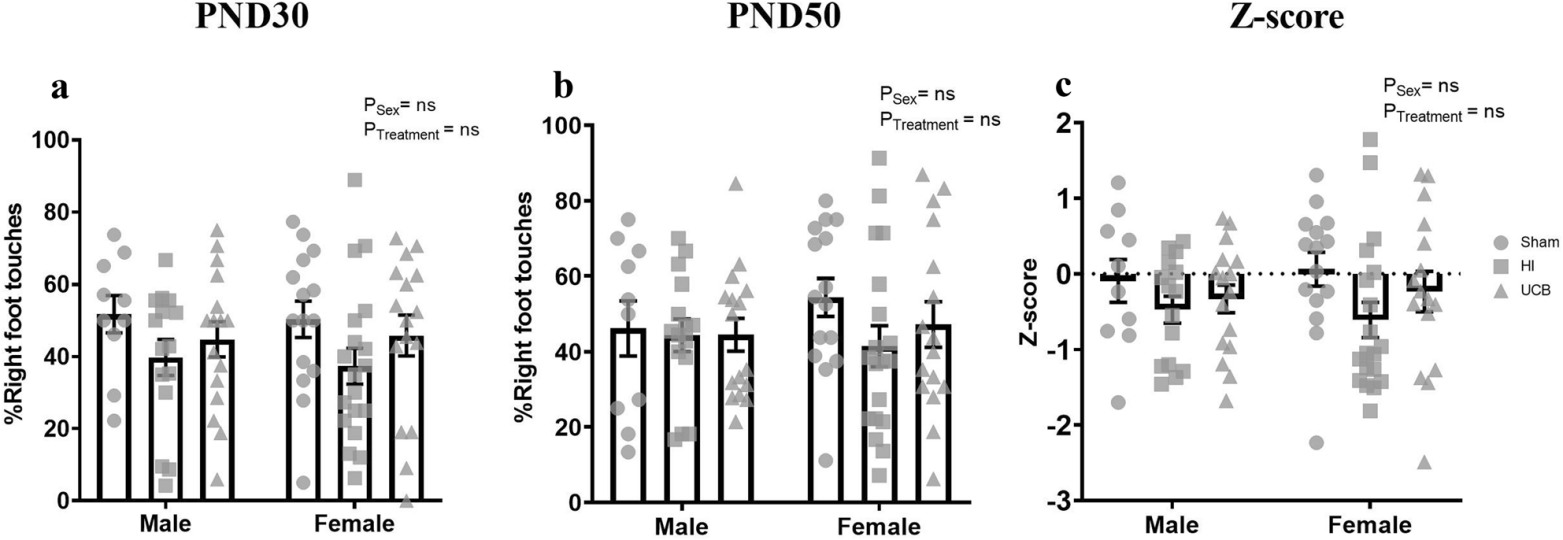
Raw and transformed scores for the cylinder test. The percentage of right foot touches on PND30 (**a**) and PND50 (**b**) was recorded. Raw scores were transformed into a cylinder test Z-score (**c**). A positive Z-score reflects an improvement in behaviour, negative Z-score reflects deficit in behaviour (In these graphs, the sham group is represented by a circle, the HI group is represented by a square and the UCB groups is represented by a triangle. Data expressed as mean ± SEM, n = 10–19 pups per group).

The cylinder test was used to investigate forelimb asymmetry at PND30 and 50. Overall there was no effect of treatment or sex at PND30 or 50 (Fig. 3a,b). At PND30, males and females showed a trend towards a reduction in right limb touch percentage in the HI group compared to sham, however this did not reach significance (Fig. 3a). UCB did not significantly increase right limb touches in both males and females. On PND50 there was no difference in males between any of the groups (Fig. 3b). Females showed a trend towards decreased right limb touches in the HI group compared to sham, however this was not significant. There was no difference in the UCB group compared to the sham and HI groups (Fig. 3b). The right touch percentage from PND30 and 50 was transformed into a z-score and combined to create an overall cylinder test Z-score. There was no significant effect of sex or treatment in this Z-score (Fig. 3c). Males showed no significant differences in Z-score between any of the groups over this time period. Females exhibited a trend towards a decrease in Z-score in the HI group compared to sham, however the UCB groups Z-score was not different from the sham and HI groups (Fig. 3c).

#### Composite behavioural Z-score

**Figure 4:**
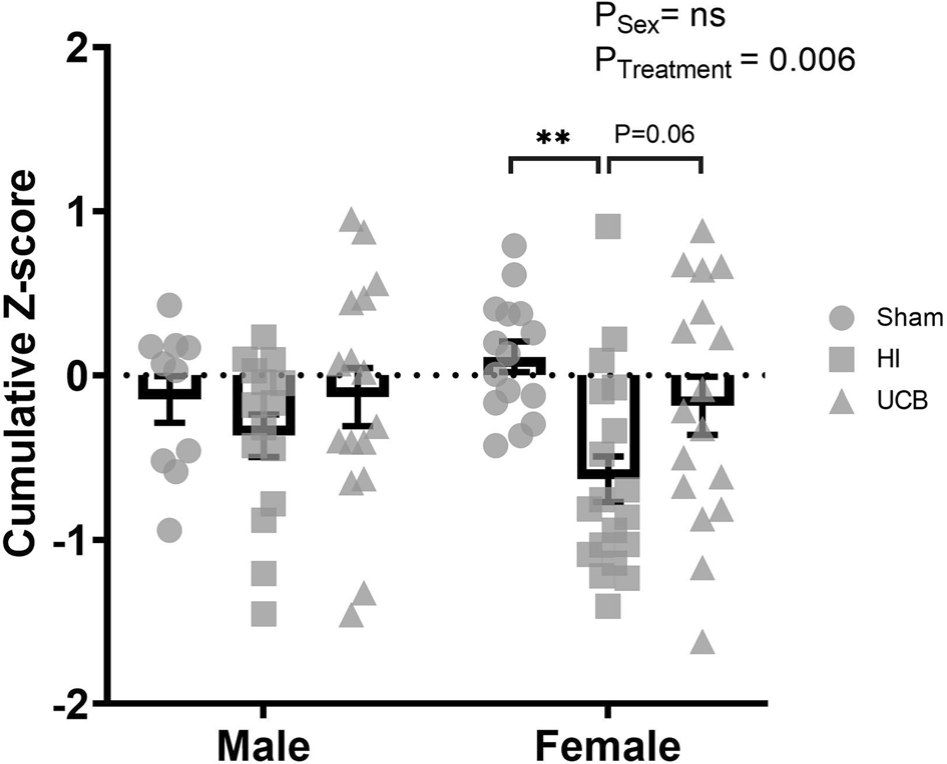
Overall composite Z-score of behavioural burden. Overall composite Z-score combining data from the negative geotaxis, novel object recognition and cylinder tests, from PND14, 30 and 50. A positive Z-score reflects an improvement in behaviour, negative Z-score reflects deficit in behaviour (In these graphs, the sham group is represented by a circle, the HI group is represented by a square and the UCB groups is represented by a triangle. Data expressed as mean ± SEM, n = 10–19 pups per group, **P < 0.01).

The Z-scores that were individually calculated for the negative geotaxis, novel object recognition and cylinder tests were combined to create a composite behavioural Z-score as a measure of overall behavioural burden^35^. Overall, there was a significant effect of treatment (Fig. 4; P = 0.006), however there was no effect of sex. In addition, there were no significant differences between any groups for behavioural burden in males. Conversely, females exhibited a significant decrease in composite Z-score in the HI group compared to sham (Fig. 4; P = 0.001), and there was a strong trend towards an increase in Z-score in the UCB group compared to HI (Fig. 4; P = 0.06).

### Brain weight and tissue loss at postnatal day 50

**Figure 5:**
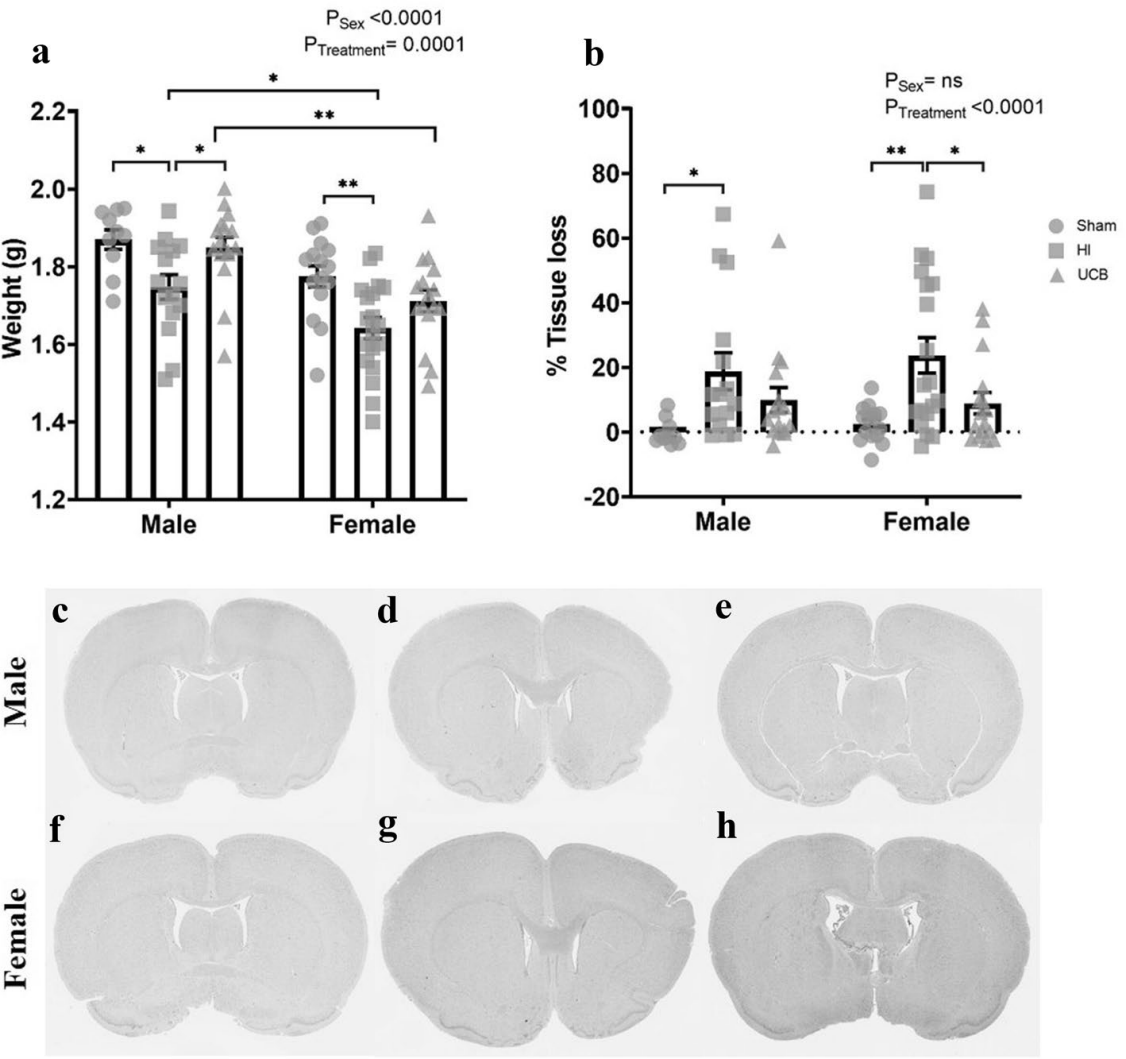
Brain weight and tissue loss at PND50. Brain weights at PND50 **(a)**. Left hemisphere area loss as a percentage of total brain area **(b)**. H&E stained representative images of males from the sham (**c**), HI (**d**) and UCB (**e**) groups and females from the sham (**f**), HI (**g**) and UCB (**h**) groups. (In these graphs, the sham group is represented by a circle, the HI group is represented by a square and the UCB groups is represented by a triangle. Data expressed as mean ± SEM, n = 10–19 pups per group, *P < 0.05, **P < 0.01).

At post-mortem (PND50), females had significantly lower brain weights overall compared to males (Fig. 5a; P < 0.0001). Further, there was a significant difference in brain weights between males and females in the HI groups (Fig. 5a; P = 0.02) and the UCB groups (Fig. 5a; P = 0.002). We also observed a significant treatment effect overall in brain weight (Fig. 5a; P = 0.0001). Further, we observed a significant decrease in brain weight in the HI group compared to sham in both males and females (Fig. 5a; P = 0.021 and 0.002 respectively). In males, brain weight was significantly increased in the UCB group compared to HI (Fig. 5a; P = 0.03), however, female brain weight was reduced with HI but not significantly improved with UCB treatment. The extent of brain injury was analysed via assessment of tissue loss in the ipsilateral hemisphere. Overall, there was a significant treatment effect (Fig. 5b; P < 0.0001), however there was no effect of sex (Fig. 5b). There was significant tissue loss in the HI group compared to sham in both males and females (Fig. 5b–d,f,g; P = 0.019 and 0.001 respectively). In addition, females showed significantly less left hemisphere tissue loss in the UCB group compared to HI (Fig. 5b,e; P = 0.029). In males, HI caused increased tissue loss and UCB did not decrease tissue loss compared to HI, however there was also no significant difference between the UCB and sham groups (Fig. 5b,h). There was no significant difference in body weight and brain:body weight ratio at PND50 between any groups (Supplementary Fig. S1 online).

### Neurons and cell death

**Figure 6:**
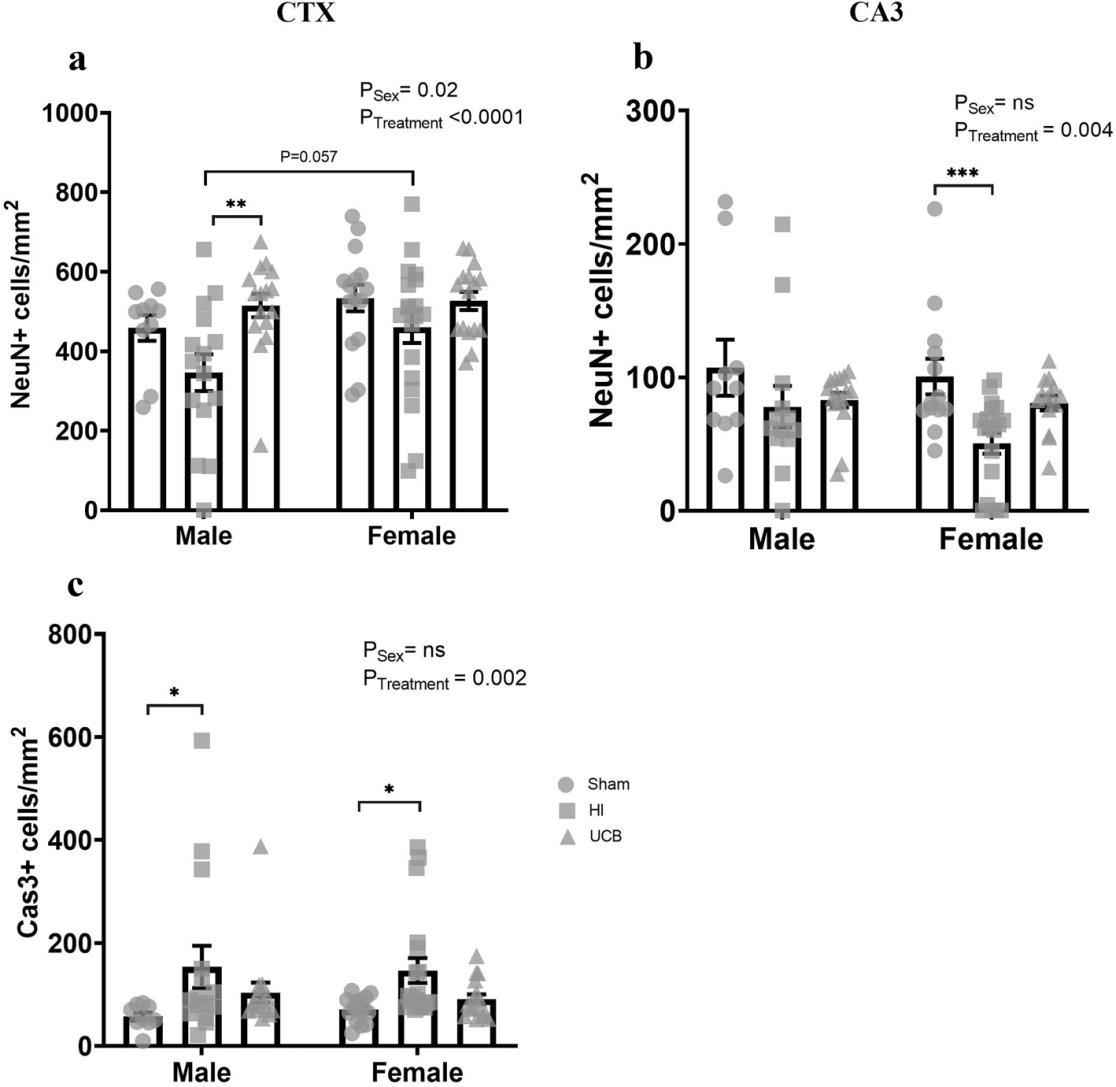
Neuron counts and cell death in the cortex and CA3 region of the hippocampus at PND50. Neuron counts determined by NeuN positive staining in the somatosensory cortex (**a**) and the CA3 region of the hippocampus (**b**) at PND50**.** Apoptotic cells determined by caspase 3 positive staining in the somatosensory cortex at PND 50 (**c**). (In these graphs, the sham group is represented by a circle, the HI group is represented by a square and the UCB groups is represented by a triangle. Data expressed as mean ± SEM, n = 10–19 pups per group, **P < 0.01, ***P < 0.001).

Neuron numbers in the somatosensory cortex (CTX) and CA3 region of the hippocampus were determined by NeuN positive staining to investigate the effects of HI on neuronal loss. In the cortex, there was an overall effect of sex with females having more neurons than males (Fig. 6a; P = 0.02), and a significant treatment effect (Fig. 6a; P < 0.0001). We also observed a trend towards a decrease in neuron number in HI males compared to HI females (Fig. 6a; P = 0.057). There was no significant difference in NeuN counts between the sham and HI group of males, although we observed a trend towards a decrease in neuron numbers in the HI group (Fig. 6a; P = 0.100). The UCB group had significantly more neurons compared to the HI group in males (Fig. 6a; P = 0.003). There was no difference observed in neuron numbers in the cortex of females (Fig. 6a). There was no significant effect of sex on neuron numbers in the CA3 region of the hippocampus, however there was a significant treatment effect (Fig. 6b; P = 0.004). In the CA3, males showed no difference in neuron number between any of the groups (Fig. 6b). Conversely, females showed a significant decrease in neuron number in the HI group compared to sham (Fig. 6b; P = 0.003), and a trend towards an increase in neuron number in the UCB group compared to HI (Fig. 6b; P = 0.107).

Apoptosis was analysed via caspase 3 (Cas3) positive staining in the somatosensory cortex. Overall, there was a significant treatment effect between groups (Fig. 6c; P = 0.002), however there was no overall difference between males and females (Fig. 6c). In both males and females, the HI group had a significant increase in apoptotic cells compared to the sham group (Fig. 6c; P = 0.033 and 0.044 respectively). Both males and females in the UCB treated groups were not significantly different from sham or HI groups (Fig. 6c).

### Neuroinflammation and astrogliosis

**Figure 7:**
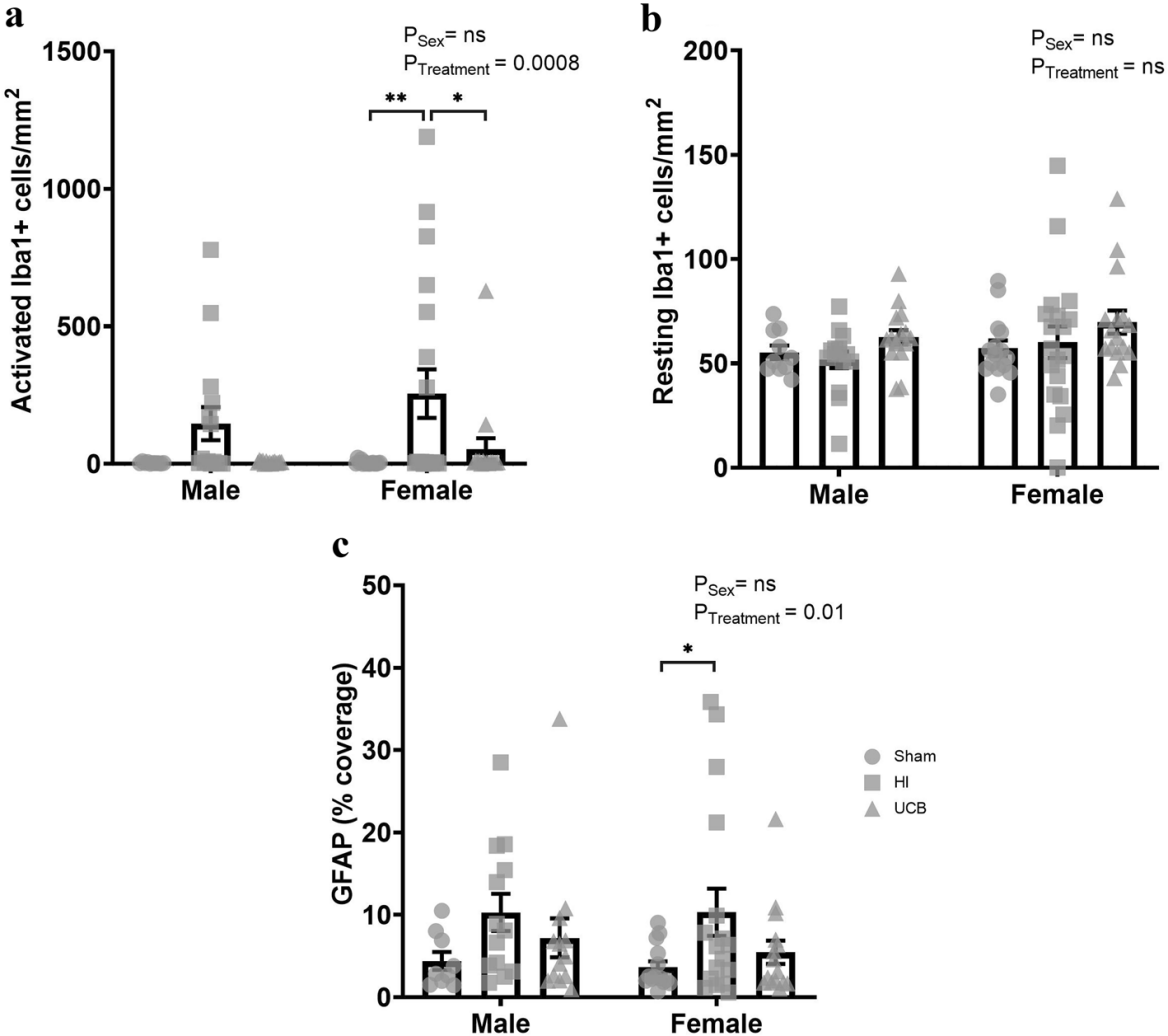
Microglia cell counts and astrocyte coverage in the cortex at PND50. Microglia cell counts determined by Iba1 positive staining where the number of activated (**a**) and resting (**b**) microglia in the somatosensory cortex was determined by cell morphology. Astrocyte coverage in the somatosensory cortex was determined via GFAP positive staining at PND50 (**c**). (In these graphs, the sham group is represented by a circle, the HI group is represented by a square and the UCB groups is represented by a triangle. Data expressed as mean ± SEM, n = 10–19 pups per group, *P < 0.05, **P < 0.01).

As a measure of neuroinflammation, microglia in the somatosensory cortex were identified with Iba1 positive staining with identification of activated and resting microglia based on cell morphology. There was a significant treatment effect seen in the number of activated microglia (Fig. 7a; P = 0.0008), however there was no effect of sex (Fig. 7a). There was no effect of treatment or sex in the number of resting microglia in the cortex (Fig. 7b). Females showed a significant increase in activated microglia in the HI group compared to sham (Fig. 7a; P = 0.002). The respective UCB group had a significant decrease in microglial activation compared to the HI group (Fig. 7a; P = 0.017). In the males, we did not observe any significant differences between groups for microglial activation (Fig. 7a). There was no change in the number of resting microglia in the cortex of either males or females (Fig. 7b).

GFAP positive staining was used to determine astrocyte coverage in the somatosensory cortex. There was an overall treatment effect in astrocyte coverage (Fig. 7c; P = 0.01), however there was no effect of sex (Fig. 7c). Astrocyte coverage was significantly increased in females in the HI group compared to sham (Fig. 7c; P = 0.05). The female UCB treated group did not exhibit a significant reduction in astrocyte coverage in females compared to the sham or HI group (Fig. 7c). There was no significant difference in astrocyte coverage in males between any groups (Fig. 7c).

## Discussion

Clinically, sex is a risk factor for perinatal morbidity and mortality, with males being more susceptible to the effects of perinatal compromise including hypoxic ischemic encephalopathy and subsequent development of CP^6–8^. This sexual dimorphism is not well understood, however hormonal regulation may play a role in how males and females respond to injury^4,14^. Previous preclinical studies using a rodent model of HI brain injury have demonstrated sexual dimorphism in cellular and molecular mechanisms and functional outcomes that are commonly affected following an asphyxic insult during late gestation or at birth^12,13,15,25,26,50–52^. This large amount of data highlights a sex-specific progression of brain injury, and, therefore, the potential for differential effects of neuroprotective treatments in female and male offspring. We, and others, have shown strong therapeutic benefits of UCB treatment following neonatal HI in rodents^35–37,39,41,43,53^ and sheep^54–58^, however the effect of sex on UCB efficacy has not been previously studied. In this study, we investigated for the first time if sex influenced the efficacy of UCB as a treatment for HI-induced neonatal brain injury. Our results demonstrate that males and females both exhibited neuropathology in response to neonatal HI, however the mechanisms involved may be different between sexes. Our results also support that UCB treatment was effective in improving neuropathology and functional outcomes in both males and females which does not exhibit a strong sexually dimorphic response. This is the first preclinical evidence that UCB is an appropriate treatment for both female and male neonates.

Currently, therapeutic hypothermia is the gold standard treatment option for term born babies with neonatal encephalopathy^59^. Therapeutic hypothermia involves lowering the core temperature to 33–34 °C for a period of 72 h to slow cellular metabolism, thus slowing the progression of injury^59,60^. While clinically it is not known if the effects of hypothermia are sexually dimorphic, a preclinical study in PND7 rats exposed to HI with subsequent hypothermia showed that this treatment was more neuroprotective in females than males^29^. A study by Smith et al. also demonstrated that hypothermia was neuroprotective in female and male rats, however the protection was observed to be task and sex dependent. Specifically, hypothermia led to improved outcomes in females for motor, auditory and spatial and non-spatial tasks, however males only showed improvements in auditory and non-spatial tasks^30^. A further study investigating hypothermia following perinatal cardiac arrest in juveniles, showed that males required a deeper level of hypothermia for neuroprotection to be equivalent to that in females^31^. This highlights the need to understand the optimised use and effects of novel therapies for perinatal HI in both males and females.

In this study we performed three behavioural tests and converted raw behavioural outcomes to standardised Z-scores. This method allowed us to create an overall score for each test and then combine data from individual tests to understand the overall behavioural response to HI. We, and others, have previously used this method of analysing behavioural burden in preclinical studies^35,43,61,62,63^, and it is also used in analyses of adult neurological conditions, including multiple sclerosis^64^ and Parkinson’s disease^65^. Results from this study demonstrated that females exhibited significant long-term behavioural burden following perinatal HI. Specifically, females showed deficits in overall vestibular function and short-term memory function at PND50. The finding of memory deficits in females was associated with a significant neuronal loss in the CA3 region of the hippocampus, which is the area of the brain responsible for memory processing^53^. The male groups showed mild deficits in short-term memory function at PND30 but not at PND50, and coincidentally did not exhibit significant neuronal loss in the hippocampus at PND50. This is in contrast with previous studies that have demonstrated that males exhibited deficits in short-term memory at PND30, whereas females did not exhibit these deficits^12,18^. In this study, we also showed no difference between males and females in terms of limb preference. This is consistent with previous studies that also showed no differences between sexes for this test^11^. Treatment with UCB improved the vestibular response and short-term memory at PND30 in males and short-term memory overall in females. In addition, when analysing the composite Z-score, UCB significantly reduced overall behavioural burden in females but not in males.

Previous preclinical studies have reported sexual dimorphism in several cellular mechanisms that are upregulated following HI. In this study, we have shown that both males and females had a significant decrease in brain weight and a significant increase in tissue loss in the ipsilateral hemisphere following HI, consistent with other studies that have reported that sex did not mediate brain tissue loss^16,20,22–24^. UCB was able to modulate brain injury, significantly increasing brain weight in males and significantly decreasing tissue loss in females compared to HI alone. We examined somatosensory cortical injury using a marker of caspase-mediated cell death and showed that both males and females had a significant increase in caspase3 positive cells, indicating similar rates of caspase-dependent cell death. Previous studies have shown that mechanisms that mediate neuronal cell death are sexually dimorphic as the neurons in males and females undergo cell death by different mechanisms^27^. Caspase-dependent apoptosis has consistently been found to be the dominant mechanism of apoptosis in females^66^. Conversely, males are more susceptible to oxidative damage than females following HI and thus more often undergo caspase-independent cell death, driven by an increase in oxidative stress^28^. We further investigated cortical injury by looking at neuron numbers in the somatosensory cortex where we showed that females were less susceptible to neuronal injury than males. This is consistent with previous studies that have suggested that males are more susceptible to neuronal excitotoxic injury due to heightened levels of testosterone^67^, however this has not been well studied.

Neuroinflammation plays a central role in mediating brain injury following a HI insult, and is known to progress for years following the initial insult^68,69^. Microglia are the resident immune cells of the brain and are activated in response to a HI insult^70,71^, with activated microglia categorised into different activation states, typically known as M1 or M2^72^. The M1, or classically activated microglia phenotype are pro-inflammatory and are associated with an increase in pro-inflammatory cytokines which contribute to the injurious response following a HI insult. M2, or alternatively activated microglia are predominantly anti-inflammatory and express receptors that work to downregulate the inflammatory response and promote tissue repair^73^. Overall, studies have shown that there are no differences between males and females in microglia activation following brain injury, however, females were shown to have a higher proportion of M2 anti-inflammatory activated microglia 3 days after an HI insult, compared to males^26^. This shift in microglia phenotype appears to be transient as a study by Al Mamun et al. showed that 30 days after HI, there was no sex difference in activated microglia phenotype^25^. In addition to microglia activation, there is a peripheral immune response following a HI injury, which involves the expression of pro and anti-inflammatory cytokines and infiltration of circulating leukocytes into the brain^69^. Females exhibited a significant increase in IL-10, an anti-inflammatory cytokine^25^. In contrast, males showed higher tumour necrosis factor (TNF) plasma concentration 3 and 30 days post insult^25,26^, which is a key mediator of the inflammatory response. Further, males showed a significant increase in lymphocyte^25^ and monocyte infiltration into the brain parenchyma^26^. In this study, inflammation was measured via microglia counts in the somatosensory cortex, 40 days after the HI insult. As previously mentioned, females were shown to have a higher proportion of M2 microglia compared to males, which indicates a reparative response by the microglia^26^. In the current study, we found that females had a significant increase in microglia activation in the HI group compared to sham, whereas males showed a tendency towards an increase in the HI group, but this was not significant. The presence of activated microglia for an extended period after a HI insult in our study may be due to the presence of M2 activated microglia that are working to activate reparative mechanisms^72^. The administration of UCB significantly reduced microglia activation in females compared to HI alone, and again there was a tendency towards a decreased response in males. Further to the microglial response, we also investigated astrocyte coverage in the somatosensory cortex and found that HI induced an increase in astrocyte coverage in the females compared to sham and a trend towards a decrease with UCB, whereas there were no significant differences in males. The effect of sex on astrocytes following perinatal HI has not been well studied, however a study that looked at sex differences in a model of adult stroke found that the astrocytes from females were more resistant to injury, such as oxidative cell death, than males^74^.

Sexual dimorphism is present in many adult and neonatal conditions, and as previously mentioned, males are more susceptible to HIE and subsequent CP^4^. Whilst it is believed that sex-specific genes and hormones drive this dimorphism, sex-specific placental adaptations may contribute to the relative risk of poor neurodevelopmental outcomes following an insult in utero^6^. It is thought that the female placenta is more equipped to adapt to an adverse intrauterine environment, thus females are less likely to be affected by an injurious insult^6,27^. Despite this clinical gender bias, preclinical rodent studies have consistently shown that both males and females are susceptible to behavioural and neuropathological deficits. Moreover, in this study, we showed that females are more vulnerable to neuroinflammation and behavioural deficits than males, contrary to clinical data. This disparity between clinical and preclinical studies may be due to the postnatal nature of our preclinical rodent studies. As the Rice-Vannucci model of HI brain injury is induced in the postnatal period, females do not have the added support of the placenta to protect against poor neurological outcomes. To overcome this limitation, future preclinical studies could use large animal models of in utero hypoxia/birth asphyxia^54,56^, where the placenta can offer support to the fetus, to mimic the human condition more closely. This, however, has restrictions as large animal models are often underpowered to examine sex differences.

A limitation of this study was that we only collected brains at PND50, and the whole brain was fixed for histological analysis. In the future, fresh brain tissue could be collected at different time points throughout the experimental period for further analysis including flow cytometry to ascertain the phenotype of activated microglia^26^, which would help us better understand the inflammatory response and potential sexual dimorphism in perinatal HI. In addition, blood should be collected to investigate the peripheral immune response, which is known to be altered following perinatal HI^36^, to determine how UCB modulates peripheral inflammation in males and females. In this study we did not analyse the contralateral side of the brain, however given the peripheral immune response could affect development in this side of the brain it would be interesting to ascertain if sex also has an effect on this uninjured side following HI injury and UCB administration. Further histopathological analysis should also include other regions of the brain including the basal ganglia and thalamus and a thorough investigation of the hippocampus, which are regions that are known to be affected in this model of HI brain injury^75,76^. In this study, the analysis was solely focused on the grey matter of the brain, not the white matter, as white matter injury is more common following preterm brain injury^77^. We have also previously found that in this model there is no significant injury to the corpus callosum as seen by looking at myelin density. An additional limitation of this study is that two different routes of administration were used for delivery of the UCB cells. We have previously compared the outcomes of the two administration routes through behavioural testing and immunohistochemistry and showed no significant difference between the intranasal (IN) and intraperitoneal (IP) treated groups in any measure and we have previously published using these mixed delivery groups^35^. Other preclinical studies have demonstrated that IP^36,43,78^ and IN^79,80^ are both effective routes for delivering cell therapies, and studies that have performed a direct comparison show variable results^81,82^. As UCB cells convey benefits via trophic mechanisms, they can work systemically, thus the administration method does not appear to significantly influence cell efficacy, however this could be considered as an extraneous variable.

## Conclusion

This study is the first to investigate the degree to which UCB is an effective therapy for both female and male neonates following a hypoxic insult. We have shown that females and males both exhibited significant injury following a HI insult in line with previous preclinical studies. Specifically, we demonstrated that females were more susceptible to long-term behavioural deficits, as well as neuroinflammation and astrogliosis. Both females and males experienced tissue loss and region-specific neuron loss. In addition, we showed, for the first time, that UCB was able to modulate aspects of injury in both females and males, although possibly via different mechanisms but do not appear to have a strong sexually dimorphic therapeutic effect. Our findings further support that UCB is an appropriate treatment for neonatal HI that will be effective in females and males.

## Methods

### Ethics approval

All experiments in this project were performed with human ethics approval from Monash Health Human Ethics Committee (12387B) and written informed consent was obtained from all participants before collection of UCB. Human tissue collection and processing was carried out in accordance with the Australian National Health and Medical Research Council guidelines. Ethics approval for animal studies was obtained from Monash Medical Centre Animal Ethics Committee A (MMCA/2015/42 & MMCA/2019/28). Animal studies were carried out in compliance with ARRIVE guidelines. All experiments were performed in accordance with the Australian National Health and Medical Research Council guidelines.

### Isolation, cryopreservation and preparation of cells

Human UCB was collected via the umbilical vein from healthy term caesarean section births (> 37 weeks), into a collection bag containing anticoagulant. The patients gave written informed consent for the collection and research use of UCB. On average, ~ 125 ml of blood was collected from each patient. To separate the mononuclear cell population, the blood was evenly separated into falcon tubes, diluted with equal amounts of phosphate buffered saline (PBS) and centrifuged for 12 min at 3100 RPM at room temperature (RT). The mononuclear cell layer was collected and washed with PBS (20 ml) and centrifuged at 800 g at RT for 5 min. Red blood cell lysis buffer, (155 Mm ammonium chloride (NH4Cl), 10 mM potassium bicarbonate (KHCO3) and 0.1 mM EDTA dissolved in distilled water) (20 ml) was added to the cell pellet to lyse any remaining red blood cells in the sample. The lysis was stopped after 3 min with media (10% fetal bovine serum (FBS), 1% antibiotic, 1% L-glutamine in DMEM/F12) (30 ml) and centrifuged at 400 g at RT for 5 min. The cells were counted using trypan blue exclusion dye with a haemocytometer. UCB cells suspended in media were transferred to cryopreservation vials and an equal volume of cryopreservation media (80% FBS containing 20% dimethyl sulfoxide (DMSO)) was added dropwise, resulting in a final concentration of 10% DMSO. Cells were cryopreserved in vials at an approximate density of 20–50 million cells/ml and were placed in a freezing container (MrFrosty, Thermo Fisher Scientific) and stored at -80˚C overnight. before being moved to liquid nitrogen for long-term storage.

### Animals

#### Animal source and housing

A total of 91 rat pups (from 13 dams) were used in this study. Rat pups were assigned to experimental groups, based on sex to ensure even distribution of males and females in each group, as well as surgery times, to control for any neuroprotective effects associated with prolonged isoflurane exposure. Pups from each litter were split amongst treatment groups to control for any litter variation and to further reduce variation, litter sizes of less than 8 pups were excluded, and were culled down if litter size exceeded 12 pups. 66 animals were exposed to HI (HI, n = 15M, 19F; UCB, n = 16M, 16F) and 25 animals received sham surgery with no HI (Sham; n = 10M, 15F).

#### Animal surgery to induce HI

A permanent unilateral carotid artery ligation was performed on PND10, followed by exposure to hypoxia as previously described^35^. Briefly, the pups were separated from their mother and placed on a 37 °C heat pad before and after surgery. The pups were anaesthetised by inhalation of 4% isoflurane which was maintained at 1–2% for the duration of the surgery, the average time under anaesthesia was 10 min, including anaesthetic induction, surgery and suturing. A small incision was made in the neck, and the left carotid artery was exteriorised before being occluded with an electrocautery device. The wound was sutured closed using 6–0 polypropylene suture and isoflurane was then stopped and bupivacaine was applied to the surgery site for pain management. The pups were returned to their dam for a 1 h recovery period. Following this, the pups that underwent artery ligation were placed in a humidified hypoxic chamber for 90 min at 8% oxygen, balanced with 92% nitrogen, and the chamber was maintained at 35–36 °C. Control pups underwent a sham surgery, in which the artery was not ligated. Following surgery, they were returned to their dam for a 1 h recovery period and were then placed on a 37 °C heating pad and exposed to normal room air for the same duration as the injury group that underwent hypoxia. Following the hypoxic treatment, the pups were returned to the dam for recovery. For the remainder of the experiment, the pups were health checked daily and weighed 3–4 times a week. Pups were weaned from their dam at PND21 and males and females were housed in separate boxes.

#### Preparation and administration of UCB cells

Rat pups received UCB cells 24 h (PND11), 72 h (PND13) and 10 days (PND20) post insult^35^. The cells were administered via either intraperitoneal injection or intranasal delivery. To prepare for delivery, cells were rapidly thawed in a 37 °C water bath. UCB cells from three different donors were pooled and washed with media to remove freeze media. The cells were counted, and viability determined using trypan blue exclusion dye; the average viability of these thawed cells was 81%. For intraperitoneal delivery, the cells were resuspended in PBS at a concentration of 5 million cells/ml. Pups received a dose of 1 million UCB cells on each cell administration day (~ 54 × 10^6^/kg on PND11, ~ 40.5 × 10^6^/kg on PND13 and ~ 27 × 10^6^/kg on PND20) via an intraperitoneal injection in a volume of 200 μl of saline using a 30-gauge insulin syringe. HI only pups received an injection of 200 μl of saline alone, using a 30-gauge insulin syringe. For intranasal delivery, cells were resuspended in PBS at a concentration of 83 million cells/ml. 30 min before cell administration, 3 μl of PBS dilute hyaluronidase was administered twice to each nostril, totalling 12 μl, to increase the permeability of the nasal mucosa. Following this, pups received a dose 1 million UCB cells on each cell administration day via intranasal administration, where 3 μl of cell suspension was administered twice to each nostril, totalling 1 million cells in 12 μl of saline. HI only pups received 12 μl of saline alone, where 3 μl was administered twice to each nostril via micropipette. As previously reported^35^, there were no statistically significant differences between the effectiveness of intraperitoneal or intranasal administration in any of the tests performed, therefore for all analyses, intraperitoneal and intranasal groups were combined.

### Behavioural testing

#### Negative geotaxis analysis

On PND14 the pups were placed head-down on a 45-degree inclined slope covered with a standard laboratory bench pad^36^. The time taken for them to turn 180 degrees was recorded, and then the time taken to walk ~ 15 cm up the slope to cross a line was also recorded. This test was performed 3 times per pup. If the pup took longer than 90 s to complete the test, or if they climbed off the board, it was considered a fail and not included in the analysis. No pups were excluded from analysis in this study.

#### Novel object recognition

The novel object recognition (NOR) test^83^ was completed on PND30 and 50 using the Topscan behavioural analysis program to track the movement of the rat inside a box, as well as the rats interaction with objects. Prior to testing, the rats were acclimatised to the room for 1–2 h, and the boxes were disinfected after each test to remove foreign smells that could influence behaviour. For the NOR test, the rats were placed in a large open box, approximately 50 × 50 cm, containing two identical objects, for 5 min where they could explore the box freely. The rats then had a 30 min rest period before being returned to the box, where one of the objects had been replaced with a novel object. They were then allowed to freely explore the box for an additional 5 min. The time spent investigating the familiar vs. the novel object in this second test was recorded and analysed. This test assesses short-term memory and exploratory behaviours of the rats, where rats with affected memory were less likely to explore the novel object.

#### Cylinder test

On PND30 and 50, the rats completed the cylinder test to analyse forelimb preference^84^. The rats were placed in a clear glass beaker and video recorded from overhead for 2 min. The cylinder was disinfected between trials to remove foreign smells that could influence behaviour. The videos were then analysed to assess, upon rearing, how often the right and left foot touched the wall of the cylinder. This test assesses if the HI injury has impaired limb use and caused asymmetric motor control.

#### Behavioural composite Z-score

As previously described^35,43,63^, all behavioural outcomes were converted into a Z-score which was compared to the Z-score generated from the sham group. Z-score was calculated by subtracting the mean test score of the control group from the individual test score, divided by the standard deviation of the control group.

$$\frac{(\mathrm{individual}\,\mathrm{ test \, score}-\mathrm{control \, mean})}{\mathrm{SD \, of \, control}}$$

For the negative geotaxis z-score data was transformed from the time to turn and time to cross the line results to generate z-scores. For the novel object recognition test z-score data from the discrimination index was used to generate z-scores. For the cylinder test z-score data from the percentage of left foot touches was used to generate z-scores. These Z-scores were summed across the included tests (negative geotaxis analysis, novel object recognition and cylinder test) to give a final cumulative score as an overall indication of behavioural deficits across both motor control and anxiety-like behaviour.

### Post-mortem and brain processing

Following behavioural testing on PND50, the rats were culled by lethal inhalation of carbon dioxide, followed by decapitation. Brains were collected and fixed in 10% formalin for 3–4 days, then processed and embedded in paraffin wax. For histological analysis, the embedded brains were sectioned in 6 µm slices.

### Histology

#### Gross brain morphology

Gross brain morphology and tissue area were assessed with Haematoxylin and Eosin (H&E, Amber Scientific, Australia). For each animal, duplicate slides at approximately 0.2 mm Bregma were examined, and data averaged across groups. Images were acquired by Aperio digital scanning (Leica Biosystems, Germany) and the volume of the left (ipsilateral to the injury) and right (contralateral to the injury) hemisphere were measured using Aperio ImageScope (Leica Biosystems, Germany). For percentage tissue loss, the difference in volume between the contralateral and ipsilateral hemispheres over the contralateral hemisphere volume was calculated, using the following formula ((volume of contralateral-volume of ipsilateral)/volume of contralateral), as previously described^85^.

#### Immunohistochemistry

For immunohistochemical analysis, brain sections were analysed in duplicates, with three fields of view analysed per region, per slide. The regions of interest examined were the somatosensory cortex (for all stains) and the CA3 region of the hippocampus (for NeuN staining only).

Microglia were identified using ionised calcium-binding adapter molecule 1 (Iba-1; 1:1000, Wako Pure Chemical Industries), apoptotic cell death was assessed using activated caspase 3 (Cas3; 1:800, R&D Systems), neuronal cell counts were assessed using NeuN (1:1000, Millipore) and astrocytes were assessed using glial fibrillary acidic protein (GFAP; 1:400, Sigma-Aldrich).

Briefly, brain sections were dewaxed in alcohol (xylene and ethanol), followed by antigen retrieval in heated citric acid buffer. Endogenous peroxidases were blocked by incubating sections with hydrogen peroxide in 50% methanol/dH_2_O. The sections were blocked with serum (Iba1: 10% normal goat serum (NGS); GFAP: 5% NGS; Cas3: 5% NGS + 2% Bovine serum albumin (BSA); NeuN: 5% NGS + 1% BSA). Slides were incubated overnight at 4 °C in the specific primary antibody. Sections were exposed to a secondary antibody for 1 h (Iba1 and Cas3: Goat anti-Rabbit IgG, 1:200; Vector Laboratories; GFAP and NeuN: Goat anti- Mouse IgG, 1:200; Vector Laboratories). Staining was visualised using 3,3-diaminobenzidine (DAB; MP Biomedicals). Slides were imaged using bright field microscopy on the Olympus BX-41 microscope. Cell counts and densitometry were performed using Image J, at 400 × magnification. Quantification of microglia cell types was achieved by classifying the microglia as either resting (branching projections protruding from the cell body) or active (no projections seen, cell body is round)^86^. Aggregates of microglia were observed as dense patches of microglia cell bodies and branching projections. All assessments were conducted on coded slides and images, with the examiner blinded to the experimental groups from which they came.

### Statistical analysis

Statistical analysis was performed using GraphPad Prism 8.0 (GraphPad Software, San Diego, USA). Statistical significance was set at a P-value of < 0.05, and all data was presented as the mean $\pm$ the standard error of the mean (SEM). Data was analysed using a two-way ANOVA with sex and treatment as variables. Multiple comparisons were performed with Sidak’s post-hoc analysis.

## Supplementary Information

Supplementary Information.
