# Supplementary material for: Umbilical cord blood therapy modulates neonatal hypoxic ischemic brain injury in both females and males
Source: Sci Rep. 2021 Aug 4;11:15788. doi: 10.1038/s41598-021-95035-1 (PMC8338979; doi:10.1038/s41598-021-95035-1)
Supplement: Supplementary file 1 — Supplementary Information. [file 41598_2021_95035_MOESM1_ESM.pdf]

**Supplementary data:**

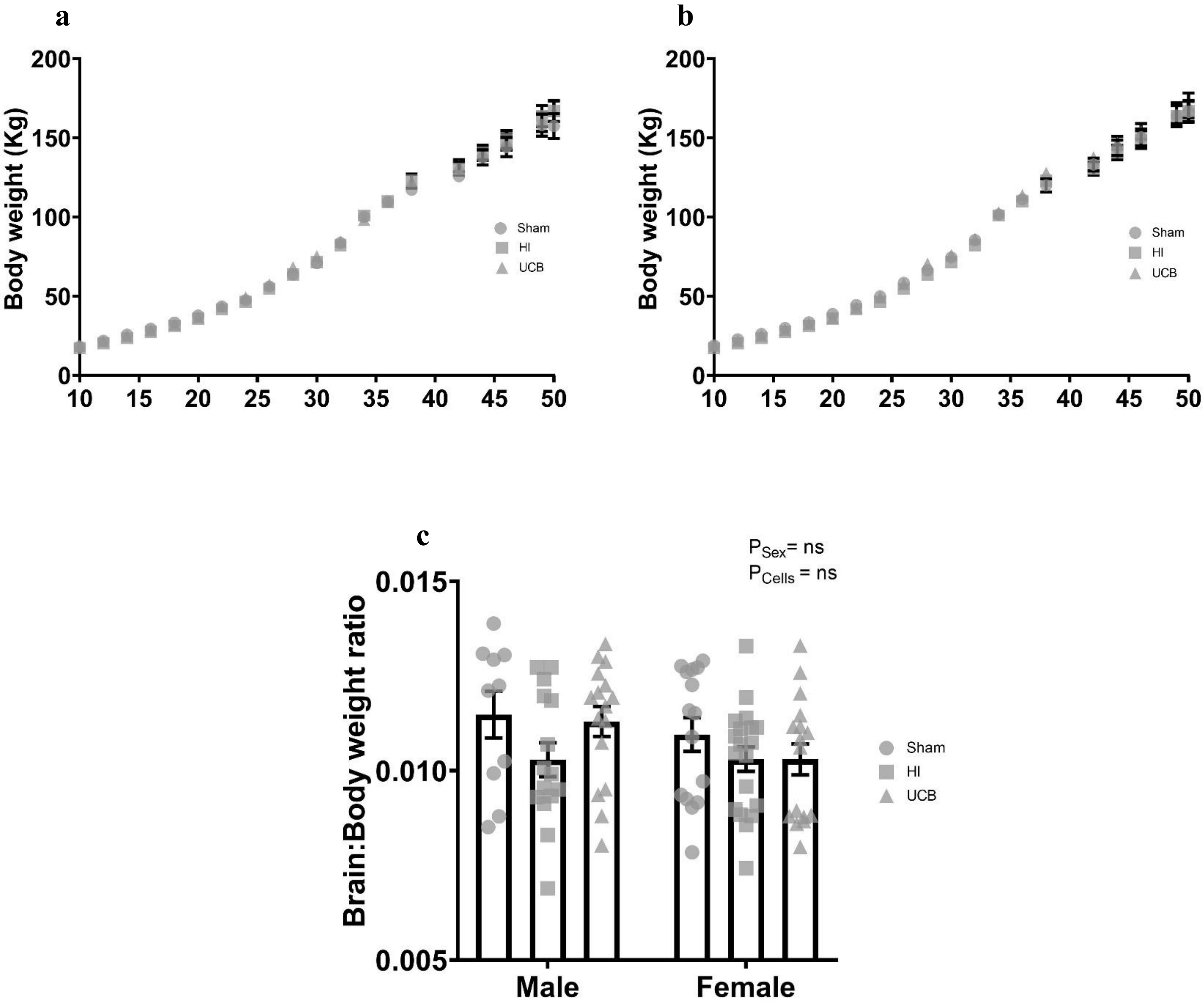


**Supplementary Figure S1 | Bodyweight and brain:bodyweight ratio**. Bodyweight in males **(a)** and females **(b)** between PND10 and PND50. Brain to body weight ratio **(c)** at post-mortem (PND50). (In these graphs, the sham group is represented by a circle, the HI group is represented by a square and the UCB groups is represented by a triangle. Data expressed as mean ± SEM, n=10-19 pups per group).
